# Hypersomnia and depressive symptoms: methodological and clinical aspects

**DOI:** 10.1186/1741-7015-11-78

**Published:** 2013-03-21

**Authors:** Yves Dauvilliers, Régis Lopez, Maurice Ohayon, Sophie Bayard

**Affiliations:** 1 Centre de référence national sur les maladies rares (narcolepsie, hypersomnie idiopathique, syndrome de Kleine-Levin), Service de Neurologie, Unité des troubles du sommeil, Hôpital Gui-de-Chauliac, 80 avenue Augustin Fliche, Montpellier cedex 5, 34295, France; 2 INSERM U1061, Université Montpellier I, Hôpital la colombiere 39, avenue charles flahault BP 34493 -pav 42 calixte cavalier, Montpellier cedex 5, 34093, France; 3 Stanford Sleep Epidemiology Research Center, Stanford University, School of Medicine, 3430 W. Bayshore Road, Palo Alto, CA, 94303, USA

**Keywords:** Depression, Mood, Sleep, Hypersomnia, Excessive daytime sleepiness, Narcolepsy

## Abstract

The associations between depressive symptoms and hypersomnia are complex and often bidirectional. Of the many disorders associated with excessive sleepiness in the general population, the most frequent are mental health disorders, particularly depression. However, most mood disorder studies addressing hypersomnia have assessed daytime sleepiness using a single response, neglecting critical and clinically relevant information about symptom severity, duration and nighttime sleep quality. Only a few studies have used objective tools such as polysomnography to directly measure both daytime and nighttime sleep propensity in depression with normal mean sleep latency and sleep duration. Hypersomnia in mood disorders, rather than a medical condition *per se*, is more a subjective sleep complaint than an objective finding. Mood symptoms have also been frequently reported in hypersomnia disorders of central origin, especially in narcolepsy. Hypocretin deficiency could be a contributing factor in this condition. Further interventional studies are needed to explore whether management of sleep complaints improves mood symptoms in hypersomnia disorders and, conversely, whether management of mood complaints improves sleep symptoms in mood disorders.

## Introduction

Excessive daytime sleepiness (EDS) is commonly assumed to result from disturbed or insufficient sleep. Various symptoms associated with EDS have been reported in the literature, indicating a multifactorial mechanism [1]. EDS is associated with many sleep disorders, such as insomnia, obstructive sleep apnea syndrome, circadian rhythm sleep disorders, and restless legs syndrome. In addition, it is a marker symptom in rare hypersomnias of central origin. However, the most frequent associations are mental health disorders and, particularly, depression [2].

A large cross-sectional study of independent risk factors associated with EDS in the general population found that current depression is a major factor, the result being unchanged after controlling for antidepressant use [3]. We recently reported a 19.5% prevalence of EDS in adults in the US [1]. However, using an EDS frequency of at least three times per week for at least three months with normal nighttime sleep duration, the prevalence was 4.7% and 1.5% in the presence of associated daytime impairment. In that study, EDS was associated with insufficient sleep, several sleep disorders, and general organic diseases in addition to psychiatric conditions (including anxiety and depressive disorders) and hypnotic and antidepressant use [1]. We previously reported strong associations between EDS and bipolar-II disorder as well as major depressive disorder (MDD) with atypical symptom features in a general elderly population [4]. Moreover, severity of EDS occurrence increased the risk of subsequent depression in elderly patients at four-year follow-up [5].

Cross-sectional studies have also repeatedly noted frequent sleep disturbances in depression [6]. Both EDS and insomnia form part of the clinical algorithm used to diagnose depression [7] and were initially considered to be a consequence of the disorder due to disturbances in monoamine activity [8]. Recent studies have suggested that sleep disorders may precede depression [5] and that non-depressed subjects with a family history of depression commonly have rapid eye movement (REM) sleep abnormalities [9]. The mechanisms by which EDS contributes to the development of depressive symptoms remain unclear. Physiological hypotheses have implicated genes associated with both the monoamine and circadian systems, related to stress-induced arousal responses and subsequent overactivity of the hypothalamic-pituitary-adrenal axis or, alternatively, mediated by increased activation of REM sleep mechanisms [10].

In this article, we describe hypersomnia assessment methods, define hypersomnia in mood disorders, and review studies that have examined mood disorder symptoms in hypersomnias of central origin.

## Definition and assessment of hypersomnias

In the literature, hypersomnia refers to a specific sleep-disorder-related diagnosis, such as narcolepsy in the International Classification of Sleep Disorders (ICSD-2) [11], or an associated symptom in various diagnostic entities, such as MDD in the Diagnostic and Statistical Manual of Mental Disorders, 4th Edition, Text Revision (DSM-IV-TR) [7]. In the latter condition, the predominant complaint is excessive sleepiness for at least one month (or less if recurrent), evidenced by either prolonged sleep episodes or daytime sleep episodes occurring almost daily.

### Excessive daytime sleepiness

The terms ‘hypersomnia,’ ‘excessive somnolence,’ ‘excessive sleepiness’ and ‘EDS’ are often used interchangeably, and EDS tends to be considered a disease or disorder. However, EDS is a symptom of a sleep disorder or other disease but not a disease *per se*[12]. Another problem with EDS is its definition, which is based mainly on one or two questions in epidemiological investigations, making for large differences across studies. The frequency, severity and duration of EDS greatly affect its prevalence, which ranges from 4% to 30% [1,12]. For example, ICSD-2 defines EDS as ‘an inability to remain fully alert or awake, or a propensity to nod or doze when sedentary, during the wakefulness portion of the sleep/wake cycle. This occurs daily or almost daily for at least three months [11].’ In clinical practice, EDS assessment usually involves self-report questionnaires. The most widely used is the Epworth Sleepiness Scale: the patient is instructed to make a probability judgment about the expectation of ‘dozing’ in eight different circumstances (for example, sitting and reading), a score above 10 being pathological [13]. Other questionnaires involve a momentary assessment of sleepiness, such as the Stanford Sleepiness Scale [14] and the Karolinska Sleepiness Scale [15].

Objective measures of EDS also exist. The Multiple Sleep Latency Test (MSLT) is the most commonly used [16]. The patient lies in bed in a darkened room in the daytime and tries to fall asleep. The patient takes five 20-minute naps, each two hours apart. Mean sleep latency <8 minutes is pathological and <5 minutes is severe. However the EDS complaint rarely correlates with objective findings on the MSLT. An alternative to the MSLT is the Maintenance Wakefulness Test (MWT), which assesses the capacity to maintain wakefulness [17]. During two or four 20- or 40-minute sessions, the patient sits in bed and attempts to remain awake. The most sensitive four-trial 40-minute MWT protocol considers latency <19 minutes as abnormal [17,18]. In rare circumstances, prolonged 24-hour continuous sleep recording is required to assess the degree of sleepiness.

### Excessive quantity of nocturnal sleep

Abnormally long nighttime sleep may be a major complaint in central hypersomnias (that is, idiopathic hypersomnia) and in the presence of associated conditions. Excessive sleep quantity varies across subjects and with age, younger individuals having longer nighttime sleep. The pathological cut-off of 9, 10, or 11 hours across the night and the 24-hour period remains controversial [19]. Based on a recent large cross-sectional telephone survey of adults in the US, we found that 6.3% of the sample reported a sleep duration of 9 hours or greater during the main sleep episode, and 8.4% reported sleeping at least 9 hours per 24-hour period [20]. In addition, 25.1% of subjects sleeping ≥9 hours per night and 40.1% of subjects sleeping ≥9 hours per 24-hour period also experienced excessive sleepiness. Moreover, excessive nighttime sleep is rarely an isolated symptom, as it frequently coexists with other organic, mental, and sleep disorders.

Because most studies report the total amount of nighttime sleep or sleep across a 24-hour period using only one or two questions, a sleep diary or actigraphy would be useful to validate the data. A sleep diary is a daily log used to record sleep-wake patterns over a period of weeks [21]. Extensively used in insomnia and circadian rhythm sleep disorders, the sleep diary allows quantifying extended nocturnal sleep and daytime naps along with available day-to-day and weekday-to-weekend variability symptoms. However, all these data are subjective, with frequent differences between the patient’s perceived nighttime and daytime sleep and objective sleep recording, and a tendency to overestimate sleep over 24 hours. Much less expensive than polysomnography (PSG), wrist actigraphy is a promising clinical tool for measuring rest–activity patterns and estimates of sleep–wake patterns over multiple days and nights [22,23]. Actigraphy permits the documentation of extended nocturnal sleep and daytime naps in clinical hypersomnia populations, but again with a tendency to overestimate sleep instead of rest, and to underestimate wake during the day [24]. The standard objective measure of nocturnal sleep is expensive in-lab PSG. PSG is typically performed during the major sleep period, often involving six to ten hours of recording, which prevents gathering information about extended nocturnal sleep or napping behavior. Prolonged and continuous 24-hour PSG under an *ad-libitum* protocol can objectively measure extended nocturnal sleep [25,26]. This procedure is rarely performed however, and only in a few sleep laboratories at high cost, in the absence of standardization or age- and gender-normative data. Nevertheless, without this procedure, it is difficult to distinguish whether hypersomnia consists of actual extended sleep or whether it simply represents an extra time spent in bed without necessarily sleeping, known as clinophilia.

### Hypersomnia associated with mood disorders

Diagnostic criteria for hypersomnia associated with mood disorders are described in three classification systems: the DSM-IV-TR [7], the ICSD2 [11], and the International Classification of Diseases (ICD-10) [27]. A complaint of excessive sleep quantity or EDS is always required. The predominant complaint of excessive sleepiness, evidenced by either prolonged or daytime sleep episodes occurring almost daily, is mandatory for a hypersomnia diagnosis related to other mental disorders under the DSM-IV. A complaint of EDS or excessive sleep is required for the diagnosis of hypersomnia not due to substance abuse or known physiological condition (nonorganic hypersomnia) under the ICSD-2. Excessive daytime sleep or sleep attacks not accounted for by inadequate sleep and/or prolonged transition to the fully aroused state upon awakening (sleep drunkenness) are criteria for non-organic hypersomnia under the ICD-10.

The ICD-10 and DSM-IV-TR include a symptom duration criterion of at least one month, and both imply either distress or impairment in social and/or occupational areas. The ICSD-2 is the only system that recommends objective measurement with PSG. However, it does not stipulate the daytime or nocturnal sleep duration, assessment procedures, or pathological cut-offs. Reduced sleep efficiency, increased sleep frequency, and increased number of awakenings together with ‘variable but often normal’ mean sleep latency on the MSLT were only proposed.

The proposed DSM-5 criteria for sleep-wake disorders nosology planned for publication this year included major changes regarding hypersomnia with elimination of the diagnosis of ‘primary hypersomnia’ in favor of ‘hypersomnia disorder,’ with concurrent specification of clinically comorbid conditions [28]. These modifications will also lead to elimination of ‘sleep disorder related to another mental disorder’ and ‘sleep disorder due to a general medical condition,’ in favor of ‘hypersomnia disorder’ with concurrent specification of clinically comorbid medical and psychiatric conditions. Sleep disorders *per se* are frequently accompanied by depression, anxiety and other cognitive mental status changes that warrant independent clinical attention and must be addressed in treatment management. As the primary users of DSM are mental health and general medical clinicians, not sleep disorder specialists, new DSM5 sleep-wake disorders criteria also included aggregation of hypersomnia disorder and narcolepsy without cataplexy, which will be distinguished from narcolepsy-cataplexy/hypocretin-1 deficiency disorder. Based on a recent cross-sectional telephone survey, a new definition of hypersomnia has been proposed in the upcoming DSM-5 revision including a frequency of ‘excessive sleepiness’ (defined by either recurrent periods of irrepressible need to sleep or to nap within the same day; recurrent naps within the same day; a nonrestorative/unrefreshing prolonged main sleep episode of nine hours or more; and/or confusional arousals-sleep drunkenness) at least three times per week for at least three months, despite normal main sleep duration lasting seven hours or longer, with significant daytime distress/impairment leading to a final prevalence of 1.5% [1].

## Definition and assessment of mood disorders

Diagnostic criteria for MDD are based on the presence of either sad mood and/or anhedonia plus four out of nine additional symptoms, including insomnia or hypersomnia [7]. Atypical depression may be considered a distinct entity or else a phase of MDD that evolves over time when the disorder becomes more chronic. To be qualified as having atypical features, a depressed patient must experience significant mood reactivity plus at least two other features, also including hypersomnia [7]. Dysthymic disorder (DD) diagnosis requires low mood present almost daily for two years plus at least two other MDD symptoms, including hypersomnia [7]. Three bipolar-related diagnoses (BD) have been individualized: BD-I, BD-II, and cyclothymia. Sleep disturbances are listed as symptoms of each BD subtype, with reduced need for sleep for manic and hypomanic episode and insomnia or hypersomnia for depressive episode. The initial description of seasonal affective disorder (SAD) stipulated frequent occurrence of hypersomnia, dysphoria, hyperphagia, and weight gain [29]. Currently, SAD is not considered a separate disorder, but instead a ‘course specifier’ that may exist in MDD or BD [7]. Mood disorders are generally diagnostically assessed with the Structured Clinical Interview for DSM-IV Axis 1 Disorder [30]. This instrument is a semi-structured interview for making standardized, reliable, and accurate diagnoses of the DSM-IV Axis 1 disorders. Self- or hetero-report questionnaires are also commonly used to quantify mood symptom severity. Self-report questionnaires include the Beck Depression Inventory (BDI-II) [31], the Hamilton Depression Rating Scale [32], the Montgomery Åsberg Rating Scale [33], the Zung Rating Scale for Depression [34], the Hospital Anxiety and Depression Scale [35], and the Inventory of Depressive Symptomatology (self-rating, IDS-SR_30_) [36]. These instruments are completed by the patients themselves. Note that the IDS also has a clinician-rated version (IDS-C_30_) [36].

However, these questionnaires should be interpreted with caution when assessing the presence and severity of depressive symptoms, higher scores being potentially without a diagnosis of mood disorder and vice versa. Similar scores using these questionnaires may also obscure important individual differences in the relative severity and frequency of somatic/affective versus cognitive symptomatology. Accordingly, we must emphasize that somatic and cognitive symptoms can be signs of physical illness and that self-report questionnaires often over-identify depression in clinical populations [37], such as patients with central hypersomnias.

## Hypersomnia associated with mood disorders

### Subjective assessment of hypersomnia

The hypersomnia symptoms associated with mood disorders are not specific and may include non-imperative EDS, long non-refreshing naps, long sleep time, and sleep inertia. EDS should be distinguished from insufficient sleep and fatigue. Fatigue is not necessarily relieved by increased sleep and may be unrelated to sleep quantity or quality. However, it is difficult to differentiate between EDS and fatigue, which may overlap considerably in mood disorders. It is also particularly difficult to differentially diagnose between idiopathic hypersomnia and less severe forms of depression (for example, dysthymia).

Albeit a diagnostic symptom for mood disorders, hypersomnia is largely understudied in this area. A recent review reported widely varying estimates of hypersomnia in MDD across age, gender and studies, ranging from 8.9% in childhood (<13 years) to 75.8% in young adulthood [2], with a higher prevalence in females. Few studies have explored the presence of hypersomnia symptoms in MDD with atypical features, with frequency varying from 24% to 56% [38,39]. The large frequency range likely reflects ambiguous definitions of hypersomnia, which vary between studies and are mostly based on a response to a single question [40-42]. The basis of hypersomnia in MDD is poorly understood; one may hypothesize that hypersomnia is related to abnormal sleep homeostasis in MDD. Interestingly, a recent high density electroencephalography study suggested that the presence of hypersomnia in MDD is associated with reduced parieto-occipital slow wave activity compared to those without hypersomnia [43].

Hypersomnia assessed with a single yes/no response (for example, ‘sleeping too much’) was found to be refractory after antidepressant intake, persisting even when other symptoms had remitted [44,45]. Hypersomnia may also predict the onset of a major depressive episode [5,42], and hypersomnia persistence after depressive symptoms have been managed may be a condition in those at risk for developing recurrent depressive episodes [41,46].

In a cohort of consecutive BD-II and MDD outpatients, ‘increased sleep’ was reported by 37.6% and 24%, respectively [38]. Another study in BD exploring hypersomnia complaints that used several questionnaires and a sleep diary for seven days between episodes showed that hypersomnia may predict the risk of future depressive symptoms, with no interaction with baseline depressive symptoms or medication use [47]. Lifetime hypomanic episodes were also associated with persistent EDS in an elderly population [4]. The frequency of hypersomnia symptoms in SAD ranged from 67% to 76% across studies [2,48-52]. However, these studies used different diagnostic tools to assess sleep complaints. One study used an original methodological approach to explore hypersomnia complaints in patients with SAD: a standard interview guide, questionnaires, and sleep diaries [53]. Results indicated longer self-reported total hours of sleep in winter than in summer but with large differences between assessment methods, and again with a tendency to overestimate total sleep time using self-reports.

### Objective assessment of hypersomnia

A few studies have used objective methods to investigate extended nocturnal sleep and EDS in mood disorders, but all reported no difference in mean sleep latency or sleep duration between patients with mood disorders and healthy controls. REM sleep was almost absent during daytime naps in the depressed phase of patients with BD [54]. Hypersomnia complaints were independent of the mean sleep latency and were related more to lack of interest and decreased energy due to depression in BD [54]. Another study revealed neither pathological sleep latencies on the MSLT nor abnormal total sleep time on prolonged PSG in patients with hypersomnia associated with mood disorders, that is, dysthymia, BD, and recurrent MDD [55]. Normal MSLT latency was also reported in dysthymic patients compared to patients with idiopathic hypersomnia and healthy controls [56]. Using the PSG and a non-conventional objective measure of EDS (that is, two 60-minute naps at 9:00 and 12:30), Vgontzas and co-workers have compared the nighttime and the objective sleepiness of drug-free patients with diagnosis of primary (idiopathic) versus psychiatric hypersomnia (that is, mood, somatoform, anxiety, and personality disorders) [57]. This group found that patients with psychiatric hypersomnia, although having complaints of EDS, showed both during the day and during the night lower sleep propensity (that is, higher sleep latency and total wake time) than patients with idiopathic hypersomnia and controls [57].

Taken together, there is no objective evidence supporting the view that patients with mood disorder have either abnormal mean sleep latency on the MSLT or objective extended nocturnal sleep. However, these patients spent a substantial amount of time in bed, acknowledged as ‘resting’ more than ‘sleeping’ (called clinophilia), with major distress and impacts on the natural course of mood disorders.

One may question whether the diagnosis of hypersomnia requires objective evidence of daytime/nighttime sleepiness or hypersomnia may be resumed as a ‘subjective sleep complaint.’ The complaint of EDS is rarely corroborated by the MSLT results, particularly in the context of associated mood disorders. Paralleling PSG-MSLT studies of hypersomnia in mood disorders, PSG evidence for insomnia often does not match self-report, and yet insomnia is currently considered an independent disorder. We do believe that hypersomnia diagnosed by a structured clinical interview as in proposed DSM-5 criteria merits clinical attention [28]. Thus, hypersomnia associated with mood disturbances may be a clinically-defined condition with significant socio-economic burden [58] that may justify a treatment. To date, no pharmacological drugs have been approved to manage hypersomnia in depressive disorders. The management bias is a concern, with potential interactions between EDS, extended nocturnal sleep complaints and drug intake. Some basic recommendations can be proposed, such as avoiding psychotropic sedative drugs (that is, benzodiazepine) and giving priority to noradrenergic antidepressant treatments for their action on the wake drive. Treatment with antidepressants, such as selective serotonin reuptake inhibitors and reuptake inhibitors of serotonin and noradrenalin, rather than psychostimulants, may be considered for patients when a mood disorder cannot be definitely ruled out as the cause of hypersomnia. However, complaints of EDS and extended nocturnal sleep may persist as refractory symptoms, despite adequate medication or behavioral and cognitive therapy that improve other symptoms. Further assessments, including objective measures of EDS, may then be useful to formally exclude an underlying central hypersomnia disorder.

## Mood symptoms associated with hypersomnia disorders

Comorbidity between hypersomnia disorders and mood symptoms, particularly depression, is frequently reported in both clinical and research settings. However, the causal relationship between the two conditions remains unclear. Hypersomnia symptoms may also be misdiagnosed as depression, because MDD symptoms according to the DSM-IV-TR are common features of hypersomnia disorders.

### Narcolepsy with cataplexy

Narcolepsy is an orphan sleep disorder (0.026% of the general population) characterized by a clinical history of EDS and abnormal manifestations of dissociated REM sleep, such as cataplexy (that is, sudden loss of muscle tone triggered by strong emotions), hypnagogic hallucinations, and sleep paralysis [59]. Narcolepsy typically starts during adolescence, a critical period of normal development and interpersonal relationship building [60]. Cataplexy is the best clinical diagnostic marker for the disease, occurring in 70% to 80% of patients. Two separate entities are individualized: narcolepsy with and without cataplexy [11]. Narcolepsy diagnosis requires nocturnal PSG recording followed by the MSLT, the latter showing a mean sleep latency <8 minutes and two or more sleep onset REM periods (SOREMPs).

For 120 years after it was identified, narcolepsy was attributed to psychiatric etiologies [61]. In the 2000s, however, it was determined that when cataplexy is present, narcolepsy is almost always caused by an immune-mediated destruction of orexin/hypocretin neurons located in the lateral hypothalamus [62,63]. Accordingly, low-undetectable cerebrospinal hypocretin-1 is observed in almost all cases of narcolepsy with cataplexy, but in only 10% to 20% in narcolepsy without cataplexy [64-67]. Studies linking loss of hypocretin neurons to human narcolepsy have spurred interest in improving the disease phenotype. Neuroanatomical studies indicate that hypocretin projections are widespread, including the cortex, the basal forebrain, limbic structures (for example, amygdala, ventral tegmental area), the thalamus, most of the brainstem (that is, locus coeruleus, raphe nucleus, and cholinergic tegmental nuclei) and the spinal cord [68]. The hypocretins act primarily as excitatory neurotransmitters to control monoaminergic and cholinergic neuron activity. Hypocretin deficiency induces a cholinergic–monoaminergic imbalance, with primary effects on vigilance as well as other functions, including mood regulation. Because hypocretins are also involved in neuroendocrine functions and stress reactions through stimulation of the hypothalamus–pituitary–adrenal axis, hypocretin deficiency *per se* may trigger mood disturbances and psychological alterations through diverse pathways.

High levels of psychopathology were frequently reported in cross-sectional studies in narcolepsy, with a high prevalence of self-reported depressive symptoms [69-72]. In a lifetime approach case–control study using DSM-IV-TR criteria, increased psychotic symptoms were found in narcolepsy with cataplexy, with no increase in depression frequency [73]. However, psychotic-like symptoms were induced by amphetamine intake and not by the disease *per se*, and symptoms were resolved when the dose was lowered or treatment changed to modafinil [73]. Another case–control study found mood disorder symptoms in one-third of narcolepsy patients. Nevertheless, no significant difference was found between patients and controls regarding the formal mood disorder diagnosis [74]. In contrast, more than half the patients had anxiety or panic attacks and 35% had an anxiety disorder [74]. Regarding depressive symptomatology, the frequency of moderate to severe symptoms ranged from 15% to 37% in narcolepsy [75-78]. Our large cross-sectional narcolepsy study found that depressive symptoms (using the BDI-II) were associated with greater EDS severity, greater alterations in physical and mental health quality of life, as well as the presence of REM sleep manifestations, such as cataplexy, hypnagogic hallucinations and sleep paralysis [76]. Although depressed patients generally tend to overscore on any scale, these data reinforced reported associations between depressive symptoms, hypnagogic hallucinations, and sleep paralysis in a population-based study [79]. A similar frequency of depressive symptoms was found in other narcoleptic populations and using other questionnaires [80-82]. Interestingly, in a five-year cohort study of patients with narcolepsy-cataplexy, mood symptoms remained relatively stable, with 25% of patients showing constant moderate to severe mood symptoms across assessments [81]. Globally, a high frequency of depressive symptoms has been systematically reported in narcolepsy with cataplexy, supporting the hypothesis of an endogenous depression [61,76,81]. This condition was previously suggested based on reduced nighttime REM sleep latency, increased REM sleep pressure and sleep fragmentation in both narcolepsy and major depression.

Although there is no cure for narcolepsy, psychostimulants such as modafinil, methylphenidate, amphetamine, and sodium oxybate are used to treat EDS and sleep attacks. Cataplexy is managed with sodium oxybate and antidepressant drugs [59]. Hence, most drugs used to manage cataplexy have mood-modifying properties. When assessing treatment effects on disease severity and psychological consequences, we observed that patients with narcolepsy with cataplexy treated with anticataplectics (mainly tricyclic agents and selective serotonin reuptake inhibitors) had more depressive symptoms and greater quality of life alterations compared to patients treated with stimulants alone [76]. Higher baseline severity of REM sleep manifestations would normally lead to higher prescriptions of anticataplectics. However, our study revealed that anticataplectics at doses prescribed for cataplexy management were ineffective in treating depressive symptoms [76].

Recent studies also support the role of specific hypocretin receptors in the modulation of depression-like behavior. A behavioral mice study after genetic or pharmacologic inhibition of hypocretin receptor signaling suggested that the hypocretin activity balance at either receptor 1 or receptor 2 produced an anti-depressant- or pro-depressant-like effect depending on the subtype activated [83].

Outside of the narcolepsy scope, some human studies have compared the levels of hypocretin-1 in the cerebrospinal fluid (CSF) in patients with mood disorders with the levels in healthy controls leading to controversial results. A reduced amplitude in diurnal variations of hypocretin-1 has been found in patients with bipolar or unipolar depression [84]. Brundin and co-workers found that suicidal patients with major depressive disorder had significantly lower CSF hypocretin levels than other suicidal patients [85]. In contrast other studies found similar CSF hypocretin-1 levels in patients with MDD and controls [86,87]. In addition, CSF hypocretin-1 levels did not correlate with the severity of depressive episode, the symptoms of depression or the number of episodes [86,87]. To our best knowledge, no studies have reported whether CSF hypocretin-1 levels are altered in depressive patients with hypersomnia or not. However, some recent results hold promise for the use of non-selective hypocretin-1 and −2 agonists and antagonists to treat several neuropsychiatric disorders, including narcolepsy, insomnia, and drug addiction. Nevertheless, potential unwanted side effects must be carefully monitored.

### Idiopathic hypersomnia

The prevalence of idiopathic hypersomnia (IH) in the general population is unknown. The age of symptom onset varies, but is frequently between 10 and 30 years [88,89]. Its pathophysiology is almost totally unknown, with no clear biological or genetic markers. Two forms of IH have been individualized: IH with and without long sleep time (LST) [11]. IH with LST is characterized by three major symptoms: (1) constant daily excessive sleepiness with unwanted and prolonged (≥1 hour) naps, less irresistible than in narcolepsy, and unrefreshing irrespective of duration; (2) long nocturnal sleep, uninterrupted and prolonged, with ≥10 hours of sleep; and (3) difficulty awakening after nighttime or daytime sleep, with frequent problems reacting adequately to external stimuli upon awakening, called ‘sleep drunkenness’ or ‘sleep inertia.’ IH without LST is characterized by isolated EDS. Daytime sleep episodes may be more irresistible, shorter, and more refreshing than in IH with LST. Nocturnal sleep in IH without LST is normal, with rare sleep inertia. However, some patients share clinical symptoms of both forms of IH, with prolonged nocturnal sleep time without sleep inertia, and others with nocturnal sleep of normal duration but with major sleep drunkenness [25,88,89]. Other symptoms such as mood changes (but not major depression), headache, and manifestations of neurovegetative impairment may be reported. IH diagnosis is based on clinical features and PSG followed by the MSLT (for IH without LST) or 24-hour continuous PSG under an *ad libitum* sleep/wake protocol (for IH with LST) to objectively confirm hypersomnia, ascertain the diagnosis and rule out other causes of hypersomnia (for example, insufficient sleep, sleep apnea syndrome, narcolepsy).

Depressive symptoms were noted in 15% to 25% using clinic-based samples of patients affected with IH, values being always higher than in the general population [55,56,76,90]. Mood changes not qualifying for a mood disorder diagnosis may precede or follow EDS onset and evolve independently. However, MDD (being an exclusion criterion) is incompatible with an IH diagnosis, unlike narcolepsy-cataplexy, which is potentially associated with mood disorders. Hence, it is unrealistic to estimate the frequency of mood disorders and depressive symptom severity in both forms of IH. However, we may suggest a bridge between hypersomnia associated with mood disorders or with depressive symptoms only and IH, with difficulty distinguishing the two conditions in some cases. Prospective studies are needed to unravel this issue, more specifically, to clarify whether mood changes in IH are consequent to difficulty adapting to the disease or whether they indicate a primary brain dysfunction.

### Behaviorally induced insufficient sleep syndrome

Behaviorally induced insufficient sleep syndrome (BIISS), or self-induced sleep restriction, is an individualized clinical entity in the ICSD-2 [11]. BIISS requires three criteria: (1) EDS complaint lasting at least three months, (2) shorter than expected habitual main sleep episode and (3) extended sleep when the habitual sleep schedule is not maintained (weekends and holidays). No epidemiological study has investigated BIISS prevalence in the general adult population. However, one study reported a 7.1% prevalence in outpatient clinic patients with a complaint of EDS [91]. From computerized self-report questionnaires, estimated BIISS prevalence was 10.4% in a large Norwegian cohort of students and associated with severe self-reported depressed mood [92]. Interestingly, the presence of BIISS was recently found to increase suicidal tendencies in Korean adolescents, even after controlling for BDI scores [93]. The limitations of these studies are the use of self-report measures and cross-sectional designs. Longitudinal approaches are needed to confirm the causal relationship between BIISS and mood disorder development.

## Conclusions

The associations between depression and daytime and nighttime sleepiness are complex and potentially bidirectional. The greatest challenges in the literature are the varying definitions of hypersomnia and the wide clinical heterogeneity of depression. Although objective tools such as PSG enable direct sleep measurement, they are unrealistic for use in large-scale population-based studies. Given the different methods used, the conflicting results are unsurprising. Nevertheless, no objective hypersomnia has been recorded consequent to mood disorders. Conversely, mood symptoms are frequently reported in hypersomnia disorders of central origin. Further interventional studies are needed to explore whether the management of sleep complaints improves mood symptoms in hypersomnia disorders and whether the management of mood complaints improves sleep symptoms in mood disorders.

## Abbreviations

BDI-II: Beck Depression Inventory; BIISS: behaviorally induced insufficient sleep syndrome; BP: bipolar disorder; BP-II: bipolar-II disorder; CSF: cerebrospinal fluid; DD: dysthymic disorder; DSM-IV-TR: Diagnostic and Statistical Manual of Mental Disorders, 4^th^ Edition, Text Revision; EDS: excessive daytime sleepiness; HAM-D: Hamilton Depression Rating Scale; ICD-10: International Classification of Disease, 10^th^ Edition; ICSD-II: International Classification of Sleep Disorders, 2^nd^ Edition; IDS: Inventory of Depressive Symptomatology; IDS-SR30: Inventory of Depressive Symptomatology (self-rating); IDS-C30: Inventory of Depressive Symptomatology (clinician rating scale); IH: idiopathic hypersomnia; LST: long sleep time; MADRS-S: self-assessment version of the Montgomery Åsberg Rating Scale; MDD: major depression disorder; MSLT: Multiple Sleep Latency Test; MWT: Maintenance Wakefulness Test; PSG: polysomnography; REM: rapid eye movement; SAD: seasonal affective disorder; SOREMPs: sleep onset REM periods; SPAQ: season patterns assessment questionnaire; BIISS: behaviorally induced insufficient sleep syndrome; ZRSD: Zung Rating Scale for Depression.

## Competing interests

SB, RL and MO declare that they have no competing interests.

YD has received speaker’s honoraria and funding for travel to conferences from UCB Pharma, Cephalon, Novartis, Jazz, and Bioprojet. YD has participated on advisory boards of Jazz, UCB Pharma, and Bioprojet. This was not an industry-supported study.

## Authors’ contributions

YD and SB performed the literature search and wrote the first draft of the manuscript. RL and MO participated in writing the paper. All authors have read and approved the final manuscript.

## Pre-publication history

The pre-publication history for this paper can be accessed here:

http://www.biomedcentral.com/1741-7015/11/78/prepub
